# Elements of cultural scripts of trauma sequelae among trauma victims in East Africa

**DOI:** 10.3389/fpsyg.2023.1206503

**Published:** 2023-10-20

**Authors:** Celestin Mutuyimana, Andreas Maercker

**Affiliations:** Division of Psychopathology and Clinical Intervention, Department of Psychology, Institute of Psychology, University of Zurich, Zurich, Switzerland

**Keywords:** cultural scripts of trauma, cultural values, East Africa, PTSD, Rwanda

## Abstract

**Background:**

As a new, unifying approach to mapping the cultural expressions of trauma sequelae, cultural scripts of trauma sequelae are empirically investigated here for the first time in a primarily qualitative study. Elements of Cultural Scripts of Trauma (CST) include the typical symptoms and appraisals of changes of those who have experienced traumatic events. These elements refer to the value orientations in the given culture.

**Aims:**

To identify post-traumatic cultural scripts’ elements and their groupings, as expressed by trauma survivors from the East African population, and to explore the cultural values that serve as a reference to such scripts’ elements.

**Methods:**

Semi-structured, in-depth interviews were conducted in nine focus groups of trauma survivors and trauma experts. Grounded theory was the basis for the content analysis, and MAXQDA was used for coding and grouping. Semi-quantitative analyses of the frequency of groupings followed.

**Results:**

The study extracted 270 elements of the cultural scripts of trauma. Three stages of cultural scripts’ elements were identified including unspeakable, heart wounds and painful scars and growth. The reported elements are only those in the three last stages and they are grouped into six categories, such as cognitive appraisals, worldview, interpersonal relationships, body-related, positive changes and changes in family interest and management, while the elements of the first stages are not codable as the survivors do not yet get the words of their expressions. The cultural values that served as a reference consisted of holding the sadness, Christianity, community reputation, solidarity, social connectedness, social cynicism, and reproductiveness, among others.

**Discussion:**

This comprehensive study with participants from several countries in East Africa collected a large number of elements of cultural scripts of trauma for this regional area. Notably, these elements were based mostly on man-made traumas, such as the genocide against the Tutsis in Rwanda. Further steps in the CST investigation are subject to future studies, such as a more systematic investigation of the relationship with cultural values and the temporal relationships within the scripts.

## 1. Introduction

In recent decades, many African countries have been involved, in one form or another, in wars, conflicts, persecution, and other forms of man-made traumatic events that have cost countless lives and brought inexpressible suffering to African populations (Musisi and Kinyanda, 2020). In particular, the East African region has experienced many man-made traumatic events in recent decades. Various civil wars in the Democratic Republic of Congo, Burundi, and Uganda, terrorist attacks in Kenya and Uganda, and the genocide against Tutsi in Rwanda involved enormous traumatic experiences that resulted in the loss of people, torture, forced displacement, injuries, and other forms of stress that have affected the biopsychosocial lives of the victims (Shook et al., 2018; Scharpf et al., 2019; Robjant et al., 2020; Mutuyimana et al., 2021; Straight et al., 2021).

Several studies have identified trauma-related disorders in East African populations, including post-traumatic stress disorder (PTSD), its complex forms, depression (Mugisha et al., 2015; Familiar et al., 2016; Bapolisi et al., 2020; Mutuyimana et al., 2023) and clinical aspects of historical trauma (Mutuyimana and Maercker, 2022). However, clinical experience has shown that survivors of trauma in East Africa may present with symptoms that differ from those in the trauma-related disorder categories. Nevertheless, diagnosis and treatment are still primarily guided by the diagnostic criteria of the Diagnostic and Statistical Manual of Mental Disorders (5th edition; DSM-5; American Psychiatric Association, 2013) and the International Classification of Diseases (ICD-11; World Health Organization, 2019), as well as assessment tools developed primarily in Western culture (Burns and Alonso-Betancourt, 2013) and not adapted to East African culture. Therefore, it is more likely that both diagnostic procedures and interventions overlook very important aspects of post-traumatic culture-related symptoms that do not fall into the Western categorization of trauma-related disorders (Pole et al., 2008; Nwoye, 2015; Maercker et al., 2019).

The literature has shown that culture influences the perception and expression of posttraumatic sequelae (Maercker and Augsburger, 2019), as trauma-related disorders may differ across cultures (Patel and Hall, 2021). However, most posttraumatic sequelae research has been conducted in Western cultures (Kirmayer and Ryder, 2016). Consequently, PTSD symptoms are assumed to be universal (Hinton and Bui, 2019), although there is evidence that diagnostic criteria for PTSD do not apply equally across cultures and that there are several trauma sequelae that do not occur in all cultures and are not captured by symptoms of PTSD or other trauma-related disorders (Patel and Hall, 2021). In addition, it has been argued that the use of sociocultural information can increase diagnostic suitability and provide a better tool for understanding human distress and suffering, with the goal of providing a culturally appropriate and effective interventions and equitable care (Alarcón et al., 2009). Moreover, the ICD-11and DSM-5 support the importance of culture in perception and expression of post traumatic experiences and cultural value orientations are shown to influence the severity and the course post traumatic sequelae (Maercker and Horn, 2013).

Based on these previous researches and clinical experience, it is noteworthy that researchers need to explore culturally specific posttraumatic sequelae that are not covered by PTSD research in the Global North (Maercker and Augsburger, 2019). To address this gap, Chentsova-Dutton and Maercker (2019) developed the concept of Cultural Scripts of Trauma (CSTs; the final ‘s’ represents its occurrence in plural).

Conceptually, CSTs can be described as post traumatic cultural-related appraisals sequentially arranged and causally interlinked (Chentsova-Dutton and Maercker, 2019). Those appraisals or symptoms are referred to as elements while the networks of such elements are considered as CSTs. To further clarify, CSTs can be understood as temporal relationships between cognitive, emotional, physical, and interpersonal elements, which form the networks (or, metaphorically, galaxies). Similar networks were explored in grief research where for example, worry and rumination form temporal relationships in which initial rumination predicts future levels of worry (Anyan et al., 2020).

In some cultures, a certain set of elements dominates, while, in others, another set of elements is particularly salient (metaphorically, they form different culturally typical “stellar constellations”). An important component of the CSTs concept is that its elements can be more or less culturally “fitting” for the culture in question and that those with a high degree of cultural fit are given particular importance. CSTs cover broad descriptions of meaning that the victims of trauma associate with the negative as well as positive post-traumatic changes within a group of people who share the same culture (Zoellner and Maercker, 2006). From that meaning, clinicians can identify attributions of traumatic sequelae that may be unhelpful to healing while appreciating the culturally normative interpretations of reactions and precipitating events (Aggarwal et al., 2013).

An important part of such scripts of trauma-related thoughts, beliefs, and interpersonal appraisals has been conceptualized by previous cognitive theories of trauma. Epstein (1991) already pointed out that there is a set of basic beliefs that are challenged by traumatic events: the beliefs that the world is benign, that the world is meaningful, that the self is worthy, and that people are trustworthy. In the same vein, McCann and Pearlman (1990) indicated that traumatic events are capable of disrupting multiple domains, including interdependence, safety, trust, beliefs about power, esteem, intimacy, and/or frames of reference. Shattered assumptions theory (Janoff-Bulman, 1992) proposed that experiencing traumatic events can change the way in which victims view themselves and the world. This theory states that negative events affect three inherent assumptions: the overall benevolence of the world, the meaningfulness of the world, and self-worth.

Although the processes described in the context of CSTs may play an important role in clinical practice and research, they have not been explored in populations exposed to traumatic events, such as in Africa. A recent study in East Africa showed that 52% of participants did not meet criteria for probable PTSD, complex PTSD, and depression, although they had other significant clinical symptoms (Mutuyimana et al., 2023). This could be due to many factors, including assessment inadequacy and mental disorder symptoms that do not fit the East African culture. Therefore, an assessment of other cultural script elements is needed. In addition, research has shown that cultural value orientations have great latent relevance in the expression of posttraumatic distress (Maercker and Hecker, 2016). Yet, there are few studies that have explored how cultural values may influence posttraumatic symptoms, especially in East Africa. Therefore, this study was conducted to answer the following questions. 1.What are the post-traumatic culture-related appraisals, referred to as CSTs elements and their groupings that are expressed by trauma survivors in East Africa? 2. How are the CSTs elements related to East African cultural values as reflected by the participants? The current study refers to East Africa as a larger geographical cultural space that includes the sub-Saharan countries between the Indian Ocean and Central Africa. According to Marsella et al.’s (2000) definition of culture, East African countries have similar cultures because they have many shared beliefs, traditions, rituals, attitudes, and languages.

## 2. Methods

Since the cultural scripts of trauma are a new concept in the literature, the study primarily used grounded theory because it is a qualitative method developed to understand phenomena about which little is known (Glaser and Strauss, 1967). An important aspect of grounded theory is that the data collection and analysis are closely linked. Consequently, the data analysis influences the ongoing data collection and vice versa (Glaser and Strauss, 1967). In this study, the data analysis influenced our subsequent selection of participants, interview questions, observation focus, and topics for further data analysis. Therefore, the study selected various trauma survivors in different focus groups and collected comprehensive information from semi-structured interviews and observations. Data obtained from clinical intervention experts and data from trauma survivors were chosen for verification (Carter et al., 2014).

### 2.1. Samples

Qualitative data were collected through focus groups and individual interviews. Because grounded theory relies on a heterogeneous sample (Glaser and Strauss, 1967), the study used different groups of trauma survivors and trauma experts. First, the data collectors conducted four focus group sessions, two different in-person focus groups with 10 Rwandan experts (five participants per session) and two via Zoom call with experts from the East African region to obtain experts’ views on the specific cultural scripts of trauma among trauma survivors. The expert panel for each group in the region consisted of eight experts, specifically two experts from Rwanda, one from Burundi, one from the Democratic Republic of Congo, one from Tanzania, one from Kenya, one from Uganda, and one from Ethiopia (see Table 1). Second, the data collectors conducted five different focus groups, composed of 64 participants, with the target population in Rwanda, including genocide survivors, former combatants, former prisoners, survivors of accidents, and a group of people who had been abused in childhood. All these groups were composed of participants with different genders, education levels, age groups, and other social variables that could increase the heterogeneity of the sample (see Table 2). Following the theoretical sampling approach, we used the information gathered during the research process to develop ideas about who might be interviewed and observed next.

**Table 1:** Demographics of the experts’ groups.

Characteristics		Frequency	Percentage
Age group	29–40	18	66.7
	41–55	9	33.3
	Minimum	29	
	Maximum	55	
	Mean	39.6	
Gender	Female	14	51.9
	Male	13	48.1
Occupation	Clinical psychologist	14	51.9
	Psychotherapist and researcher	5	18.5
	Medical doctor	3	11.1
	Psychiatric nurse	2	7.4
	Social worker	2	7.4
	Child protection specialist	1	3.7
Education level	PhD	4	14.8
	Master’s	18	66.7
	Bachelor’s	5	18.5
Country	Rwanda	16	59.3
	Burundi	2	7.4
	DRC	2	7.4
	Tanzania	2	7.4
	Uganda	2	7.4
	Kenya	2	7.4
	Ethiopia	1	3.7
Type of meeting	In-person focus group	10	37.0
	Zoom focus group	12	44.4
	Individual session	5	18.5

DRC, Democratic Republic of Congo.

**Table 2:** Demographics of the lay participants.

Characteristics		*N*	Percentage	
Age group	25–40	19	29.7	
	41–60	38	59.4	
	60+	7	10.9	
	Minimum	25		
	Maximum	81		
	Mean	48.6		
Gender	Female	38	59.4	
	Male	26	40.6	
Marital status	Married	42	65.6	
	Single	13	20.3	
	Widowed	9	14.1	
Education level	No studies	8	12.5	
	Primary school	27	42.2	
	High school	22	34.4	
	Bachelor’s	7	10.9	
Group of trauma survivors	Genocide survivors	20	31.3	
	Childhood trauma survivors	10	15.6	
	Ex-combatants	8	12.5	
	Ex-prisoners	19	29.7	
	Accident survivors	7	10.9	
Country	Rwanda	59	92.2	
	DRC	1	1.6	
	Tanzania	1	1.6	
	Burundi	1	1.6	
	Kenya	1	1.6	
	Uganda	1	1.6	
Type of meeting	Focus group	59	92.2	
	Individual	5	7.8	

DRC, Democratic Republic of Congo.

Therefore, we also conducted five additional individual interviews by telephone with trauma survivors (one per country) in the East African countries named above. In addition, five individual interviews were conducted with Rwandan licensed psychotherapists currently working in psychotherapy clinics with experience in clinical interventions and psychotherapy in the East African context. These new data were used to determine whether additional relevant categories might emerge, whether the categories were well established, and whether the relationships among the categories were fully developed (Glaser and Strauss, 1967).

### 2.2. Data collection

This study used multiple methods, as suggested by Bluhm et al. (2011): semi-structured in-depth interviews, observations, and memos. Following an orienting theoretical perspective (Locke, 2002), the in-depth interviews were based on semi-structured interview guides derived from the existing literature on CSTs. These interview guides covered the aspects of trauma-related appraisals in worldview, cognition, interpersonal relationships, and stress-related growth and cultural values that may be related to these appraisals (see Appendix 1).

The questions for the focus groups of experts and trauma survivors were similar. The only difference was that the experts had to talk about their patients’ or clients’ experiences, while the survivors had to talk about their own experiences. The focus group interviews were conducted by four clinical psychologists and the first author. Two of the data collectors were master’s students in clinical psychology, while the others were master’s graduates and licensed clinical psychologists who worked with the Community Healing and Reconciliation Program and had experience with qualitative data collection. The experts from East African countries who participated in the study were purposively selected based on their presentation at the forensic summer school held in September 2022 organized by the University of Rwanda and the University of Hamburg. The same was true for the trauma survivors as we selected groups that had experienced different traumatic events but were also able and willing to talk about their experiences.

It was important that mutual trust prevailed in the focus groups. Therefore, no recordings were made, but, with the consent of the participants, extensive notes of the group process were taken, and these form the basis of the following analyses. Each session lasted between 3 and 4 h. After each session, the interviewers and the team of data collectors held a debriefing session of 1–2 h to review all the data collected during the interview and observations with a fresh mind and write them down in a readable format.

### 2.3. Data analysis

The data, transcribed in the Kinyarwanda language, were coded using the MAXQDA software (version 2022). They were analyzed using content analysis, following the steps of the grounding theory coding process, such as open, axial, and selective coding. The data were reviewed by a coding unit of two independent raters from a pool of five raters, all with clinical psychology education, and subsequently the two coders met for joint coding sessions (Corbin and Strauss, 2008). In their meeting, they compared group codes and discussed discrepancies until a consensus was reached on which code was appropriate. The quality of the coding system was confirmed by testing for interrater reliability using Krippendorff’s alpha (Krippendorff, 2013), and the overall agreement percentage was 0.88, indicating high interrater reliability of our category system.

Finally, we conducted member checks to give voice to our participants (Bluhm et al., 2011) and to ensure that the categories derived in this study were indeed based on the data. At the end of the process, the results were returned to the five experts and five focus group participants to obtain feedback on the identified categories. Participants were selected based on their English proficiency and social skills as well as their experience working with trauma survivors. Their ideas and comments were integrated into the category system.

To illustrate the results better, semi-quantitative frequencies were obtained using the MAXQDA Code Relations Browser (CRB). To test the closeness and similarity of the categories, a code map was designed using MAXQDA. This type of analysis indicates each circle that symbolizes a code, with the distances between two codes representing how similarly the codes have been applied in the data. The more two codes co-occur, the more similar they are in terms of their use in the data and the closer they are placed together on the map (VERBI Software, 2022).

## 3. Results

The content analysis followed the objectives of this study and found 270 cultural script of trauma elements categorized into 27 sub-categories and seven categories. One category includes elements of the unspeakable. These are the post-traumatic experiences that survivors cannot talk about and that require a social, familial, and political space to be processed and verbalized. The unspeakable can be regarded as first stage in the development of trauma sequelae. The five categories include the elements that survivors can talk about and that are perceived by trauma survivors as either heart wounds or painful scars, which represent the second stage in the development of trauma sequelae disorders. These categories include cognitive appraisals, worldview, interpersonal relationships, physical changes, and changes in family interest and management. The final category includes elements of positive change or growth resulting from the traumatic experience. Positive change represents the third stage in the development of trauma sequelae. Selected quotations from participants for each sub-category are provided in Table 3. The occurrence of codes in categories and sub-categories is presented in Table 4.

**Table 3:** Categories and sub-categories with selected examples of the semantic units of the content analysis.

	Labels	Examples of semantic units
Categories and sub-categories	Cognitive appraisal elements	
	Helplessness	“I rarely feel in control of my life”
	Negative perception of self	“Everyone knows what happened to me. I feel that I am dirty inside and I lost my dignity”
	Feeling bitterness	“I feel guilty for the crimes we committed and for their long-lasting consequences”
	Revengeful thoughts	“… but I hate them and I wanted to take revenge … it is a shame that it is not allowed in our culture”
	Insecurity and emotional fragility	“Since then I feel insecure, instable, stressed without peace inside”
Category and sub-categories	Worldview elements	
	Negative changes of beliefs	“After my accident, I realized that this world is not our home, that it can be left at any time … People are too bad. I cannot believe that I was betrayed by my friend … even though I was innocent”
	Hypersensitivity to incident	“I think it is necessary to report any suspicious incident, no matter how small, in a timely manner … no joke”
	Hedonism	“I cannot save money. What I have I use for my pleasure, I do not know what will happen tomorrow”
Category and sub-categories	Interpersonal relationship elements	
	Selective relationship	“I cannot make any strong relationship with a person who does not share the same traumatic experiences”
	Feeling of rejection	“I feel our manager and co-workers are not friendly to me just because I have a background of living in the orphanage”
	Bragging about achievements	“I invite my enemies to special occasions: graduation, wedding … I want them to see that we are still alive”
	Mistrust	“Friends, priests, leaders, neighbors, all were ready to kill us. How can I trust a human being?”
	Need for care and consideration	“We sacrifice our lives for the country and for the people. People need to appreciate what we have done for them”
	Valorization of personal relationships	“… did not expect. … I realized that you cannot survive without people”
Category and sub-categories	Body-related elements	
	Others	“Loss of consciousness, unusual smell, tingling”
	Pain	Headache, backache
	Disease	Sleep disorders, eye disease
Category and sub-categories	Growth	
	Wisdom	“I can say that I have become wise … bad and good things taught me how to behave in any situation”
	Human rights activism	“Now, wherever I am, I fight against injustice”
	Self-determination	“With the hurtful experience we come to the strengths. We know today that nothing is impossible if you set clear goals”
	Gratitude and kindness	“… since then I learn to be grateful and kind in every case”
	Spiritual growth	“Before my accident I scoffed at the power of Jesus, but now I am the witness of his grace”
Category and sub-categories	Change in family interest and management elements	
	The desire to give birth to many children	“I remained alone in my family, I felt hopelessness until I gave birth to the firstborn. … oh, now I wish to give birth to more …”
	Difficulties in family resource management	“I know I’m misusing resources, and that leads to misunderstandings with my husband …”
	Loss of family harmony	“I am no longer enthusiastic. I am aggressive toward my children and my partner”
	Loss of interest in marriage	“I do not want to give birth to other people who are only in the world to suffer”
Category	Unspeakable	“I feel something that I have no means to explain”

**Table 4:** Occurrences of codes in categories in the different focus groups.

Categories	Survivor of genocide	Accident survivors	CHT survivors	Ex-combatants	Ex-prisoners	Experts	Total
Cognitive	55	4	10	4	26	10	109
Learned helplessness	6	0	1	0	3	1	11
Negative perception of self	14	1	3	0	7	3	28
Feeling bitterness	12	2	4	3	9	4	34
Revengeful thoughts	16	0	0	0	4	2	22
Insecurity & emotional fragility	7	1	2	1	3	0	14
Interpersonal relationship	14	5	14	7	9	5	54
Selective relationship	3	0	5	0	0	0	8
Feeling of rejection	2	1	6	0	3	0	12
Bragging about achievements	1	0	1	0	0	1	3
Mistrust	7	0	1	0	4	2	14
Need of care and consideration	0	0	0	5	0	1	6
Valorization of personal relationships	1	4	1	2	2	1	11
Growth	8	3	9	0	8	5	33
Wisdom	1	0	1	0	7	2	11
Human rights activism	2	1	0	0	0	0	3
Self-determination	1	1	5	0	0	1	8
Gratitude & kindness	0	0	2	0	0	2	4
Spritual growth	4	1	1	0	1	0	7
Worldview	10	3	2	6	3	7	31
Negative changes of beliefs	9	2	1	2	0	3	17
Hypersensitivity to incident	1	0	0	0	3	3	7
Hedonism	0	1	1	4	0	1	7
Body-related disorders	10	3	4	0	2	2	21
Pain	5	1	0	0	0	0	6
Disease	5	1	1	0	2	2	11
Other body related disorders	5	1	3	0	0	0	9
Change in family interest and management	5	0	5	0	0	10	19
Need to have many children	5	0	3	0	0	3	11
Difficulities in family resource management	5	0	1	0	0	2	8
Loss of family harmony	5	0	0	0	0	3	8
Loss of interest in marriage	5	0	1	0	0	2	8
Unspeakable	5	0	1	0	0	0	3

This table was obtained by using MAXQDA Code Matrix Browser (CMB). CHTS, Childhood Trauma Survivors.

### 3.1. Cultural scripts elements of the first stage: unspeakable

#### 3.1.1. Unspeakable

Many participants testified that they have various trauma-related experiences that make them suffer, but they have no concept to explain these experiences in any language “Ibitavugwa” in Kinyarwanda or “Isiyosemeka” in Swahili. For them, this is the first stage of post-traumatic appraisals. Participants metaphorically compare the unspeakable experiences to an immature abscess that needs to be treated with lotion and other medical agents in order to mature. This medical treatment is likened to a safe and trusted space for expression, social support, and social recognition. These community interventions help to verbalize the unspeakable experiences metaphorically explained as a burst abscess, and then reach the second stage of heart wounds translated as “ibikomere” in Kinyarwanda or “majeraha ya moyo” in Swahili. If these heart wounds are not treated well, they leave painful scars “inkovu ziryana” in Kinyarwanda or “Nkovu chungu” in Swahili. However, participants said that good treatment of heart wounds and painful scars together with other community and pollical interventions contributes to positive changes and resilience, which form the third stage of post-traumatic appraisals “ukwiyubaka” in Kinyarwanda or “kujijenga upya” in Swahili. They said that the hurtful experiences of the unspeakable experiences are felt only by the trauma survivors. They consider them as a burden of life or a cross to bear. Therefore, they asked for continuous social support from the community to help them reach the next stages.

### 3.2. Cultural scripts elements of the second stage: heart wounds or painful scars

#### 3.2.1. Cognitive appraisal elements

The following are the seven main themes in the total of 109 single elements. The selected example for each theme is mentioned in Table 3.

##### 3.2.1.1. Negative perception of self

Depending on the type of traumatic exposure, the victims of East Africa experienced a negative perception of their image. The survivors of the genocide and war stated that their self-image had been destroyed by the atrocities and shameful acts committed against them, like sexual violence, harassment, extermination of their human and economic resources, and negative labels like snakes and cockroaches, among others. Victims of childhood trauma who grew up in orphanages indicated that their self-image had been damaged through devaluation and bullying by their caregivers. Former prisoners who committed atrocities such as killing people stated that they had become like animals, cruel and outcast from the community. Conversely, the survivors of accidents who had lost body parts feel that their beauty and reputation are no longer recognized. They identified feeling unhuman, without dignity, dirty inside, not loving themselves, and inner psychological pain as the indicators of their negative perception of self.

##### 3.2.1.2. Feeling of bitterness

The trauma survivors explained that the feeling of bitterness was very common after traumatic exposure. They linked such a feeling with the sadness related to different types of loss, including loss of people, loss of parts of the body, loss of the time spent in prison or in institutional care, and loss of physical well-being due to chronic diseases like HIV. The feeling of loss associated with post-traumatic critical living conditions, like poverty, unemployment, living alone, and preoccupation with past traumatic memories, enforces their feelings of loneliness, guilt, and desperation without the meaning of life. The feeling of bitterness manifests itself in a lack of self-care and plans for the future.

##### 3.2.1.3. Helplessness

Similar to the feeling of bitterness, the trauma survivors showed that thinking about their inability to recuperate their lost properties and their important people, associated with the decrease of people’ compassion, pushes them to feel left behind by life and by the people around them. They stated that they can find no way to deal with their daily problems after passing through the difficult moments and losing so many important things in life. They said that sometimes they think that it would be better to die than to live in the world without social, economic, and mutual support. Feeling helpless induces the symptoms of deep sorrow, heart wound, or, for some survivors like ex-prisoners, feelings of a bounded heart or heavy heart.

##### 3.2.1.4. Revengeful thoughts

The survivors testified that atrocities and crimes committed against survivors of various traumatic events in East Africa were in most cases committed not by outsiders but by neighbors and relatives or people who knew each other, for example the genocide against Tutsi in Rwanda or various wars in other countries, such as the Democratic Republic of Congo, Burundi, the Soudan, and Uganda.

The revengeful thoughts were reinforced by the proximity of survivors and perpetrators in a culture that supports revenge, such as in Soudan, or in a culture that prohibits revenge, for instance in Rwanda and Burundi. According to the trauma survivors, when revenge is not executed, it is always a source of deep sorrow and guilt.

##### 3.2.1.5. Insecurity and emotional fragility

The trauma survivors expressed that, most of the time, they feel insecure in the environment in which they might be living. This is the case for genocide survivors, who always felt that they could be attacked by their perpetrators, and perpetrators, who also felt insecure because the survivors could seek revenge at any time. In the same vein, survivors of childhood trauma stated that they are insecure and instable in their relationships. This insecurity is usually associated with emotional fragility manifested in maladaptive coping strategies. For example, some survivors stated that, when they have problems in their family or at work, although they might be minor problems that could be solved immediately, they are not able to ask their partner or employer about them because they fear being physically and emotionally abused or kicked out. Instead, they prefer to hide to cry and feel released. However, they added that this provokes heart wounds and does not procure any sustainable response to their problem.

##### 3.2.1.6. Reference to the cultural values of cognitive appraisals

The trauma survivors have an associated negative perception of self and feelings of bitterness and helplessness as their culture promotes the values of not showing suffering and maintaining self-reputation. For example, the survivors expressed that sharing heart pain is considered to be a sign of emotional weakness, and their feelings continue to be bitter, which in turn provokes a negative self-image and feeling of rejection by the surrounding community members. They indicated that it is more important to maintain their reputation and integrity than to talk about their inner pain. Furthermore, feelings of bitterness and helplessness may be induced and people feel rejected as their reputation in the community is no longer recognized positively due to the crime committed (in the case of ex-prisoners), neglect of their intellectual and moral capacity (in the case of children who lived in an orphanage), or loss of wealth (in the case of genocide survivors). Some trauma survivors explained that revengeful thoughts are due to the culture that prohibits revenge, while others said that these thoughts were motivated by some sub-cultures that consider revenge to be a value to promote.

#### 3.2.2. Worldview elements

The participants in this study announced that they perceive the world differently since experiencing traumatic events. Some of them said that previously they had not been aware of the black side of the world. Below, we explain the main themes identified in their interviews on worldview perceptions.

##### 3.2.2.1. Negative changes of beliefs

The most challenged beliefs were the beliefs about the protection of God, the kindness of human beings, and the benevolence of the world. Around 98% of the study participants believed in the power of God and most of the time were aware that He is almighty and can save them at difficult moments. During the war or genocide, many of the survivors flew into churches thinking that nobody could attack them; however, some were injured, abused, or witnessed the death of their people while praying. Hence, they doubted the power of God. The second belief challenged was the benevolence of the world. The living style in East African countries was dictated by cooperation, helping each other, and generosity. For them, brotherhood was their culture. They thought naively that everyone in their community was at least kind, but this belief was challenged by being hunted and killed by their neighbors and relatives, betrayed by their trusted persons, or not helped by people whom they expected to help them.

##### 3.2.2.2. Hypersensitivity to incidents

According to the trauma survivors, most of the events that traumatized people in East Africa started as small and easily manageable problems but suddenly turned into a complex and most horrible situation. Conversely, involvement in such horrible events was viewed as a simple initiative of fighting for their rights or thinking that it is a way of manifesting patriotism (e.g., genocide perpetrators). All of these traumatic events adversely affected the lives of the survivors and perpetrators in such a way that they became hypersensitive and more suspicious about anything. This led them to take every situation that they suspected to hurt their life very seriously; hence, in some instances, they show exaggerated responses to minor problems.

This hypersensitivity is found in everyday life, with some people needing to have a stock of food and being cautious in every single decision that involves personal responsibility, like signatures, and others believing in the importance of putting security checkpoints everywhere and having the phone details of people responsible for security because they may need them at any time.

##### 3.2.2.3. Hedonism

According to the participants, the traumatic events that happened in these countries were sudden, multiple, and unpredictable. Hence, some of the survivors lost their feeling of stability. They think that their life is full of sudden threatening events. Therefore, they have decided to enjoy the life that they have today because they are not sure of what will happen in the near future. This view has affected their psychosocial and economic achievements, but they consider it to be a way of coping with the grief of having lost many things.

##### 3.2.2.4. Reference to cultural values for the worldview elements

The participants explained that the experienced worldview perceptions were induced by various cultural values. First, the loss of a culture of solidarity and mistrust was the factor that caused trauma survivors to feel that there is no need to believe in people because they are no longer helpful at all. Second, the culture of Christianity, before the traumatic events, involved a cultural belief in Almighty God for many survivors; however, just after the traumatic events, their faith was challenged because they found that God was not able to protect them and/or to support them in the current critical conditions. Thinking about this naïve attitude of God in the face of their problems was related to a negative perception of the world and God. Lastly, survivors indicated that it is part of their culture to learn from their mistakes and to use effort, knowledge, careful planning, and other resources to achieve a positive outcome. This culture of reward for application was associated with hypersensitivity to incidents because the survivors did want to not return to the past traumatic experience.

#### 3.2.3. Interpersonal relationship elements

##### 3.2.3.1. Mistrust

The history of atrocities, such as genocide and wars, that has devastated the East African region is marked by betrayal committed by people who were previously close friends. This has led the victims to believe that all people are unfaithful because even their so-called friends can suddenly become enemies. Consequently, trauma survivors are afraid and reluctant to share their emotional and psychological pain with others. This may increase somatic disorders such as headaches and depressed mood because they feel lonely. When it comes to marriage, such people suffering from distrust have difficulty finding a partner, and, even when they marry, they always doubt their spouse. Consequently, this can affect the marital relationship.

##### 3.2.3.2. Selective relationships

The participants in this study indicated that, after traumatic events, they felt unable to establish a relationship with people who did not share their traumatic experience. According to them, this is because the trauma sequelae cannot be valued by someone outside of the traumatic context. Consequently, this attitude increased the level of mistrust, and the lack of social support was able to provoke other mental disorders.

##### 3.2.3.3. Feeling of rejection

Some participants showed that they felt rejected by other members of the community because of their background. This is, for example, the case of the ex-prisoners, who are not welcomed in the community and labeled as genocidaires even after finishing their sentence. The same applies to the children who lived in the orphanages, who are most of the time considered as impolite and without cultural values due to their background.

##### 3.2.3.4. Bragging about achievements

The survivors testified that they brag about their successes, like graduating from high school, finding a job, buying a car, having a wedding ceremony, and so on, to their perpetrators. They do their best to show their perpetrators that they are wealthy, smart, and happier than they are. The survivors said that they seem to engage in prosocial activities for their perpetrators, for example paying for their child’s tuition, giving them food, or paying their medical bills, just to show them that, even though they tried to betray them, they continue to be less successful people who need their support. Such kinds of trauma-related changes, as reported by the survivors, can be a factor of resentment, anger, and depressed mood because they can only abuse their resources for their honor and live a life of competition with their perpetrators or unconsciously with their neighbors.

##### 3.2.3.5. Need for care and consideration

This is one of the most visible changes in the participants who have experienced childhood trauma in orphanages and former combatants. Children who grew up in orphanages said they had never met anyone in their lives who cared about them. Most of the time they were neglected and abused. Therefore, they always feel the need to be cared for, respected, and appreciated in every activity. They said that they are sad because, even after reintegration into the community, they try their best to be valued like other people, but they are mostly disappointed because people always associate any simple failure with their past experiences in the orphanage. Conversely, the interviewed ex-combatants believe that they are the people who gave everything, including their lives, to save the people and the country. Some of these participants entered military service when they were too young, and they testified that they left behind their parents’ care, their wives, and their zest for life to save lives. Therefore, they said, now is the time for everyone to take care of them and reward and recognize their achievements. However, according to them, people’s attitudes are different, leading to feelings of sadness, helplessness, and valuelessness.

##### 3.2.3.6. Valorization of personal relationships

All the types of survivors interviewed in this study indicated that they became aware of the value of a good personal relationship after experiencing trauma.

They learned that they were supported by their friends, relatives, colleagues, or neighbors during wars, genocide, prisons, accidents, or childhood trauma. Therefore, good interpersonal relationships are of great value to them in life.

##### 3.2.3.7. Reference to cultural values for interpersonal relationships elements

According to the trauma survivors, trauma-induced perceptions of positive and negative interpersonal relationships were connected to the following cultural beliefs. For them, first, the perception of mistrust was related to the fact that there is a high level of hypocrisy and social cynicism among people, which is culturally known. Second, the trauma survivors showed that community reputation is one cultural value that can help someone to deal with the negative feelings. However, the trauma survivors did not feel recognized by the community either for their past benevolence (in the case of ex-combatants) or for their trauma-related suffering (in the case of genocide survivors) or for their positive changes after traumatic events (in the cases of ex-prisoners and childhood trauma survivors). Last, the cultural value of social connectedness was related to positive interpersonal relationships. The culture of mutual support that continues to be maintained by some people despite life’s challenges was able to induce compassion that was not present before the traumatic event.

#### 3.2.4. Body-related change elements

The participants in this study reported suffering from skin aches, temporary loss of consciousness, a feeling of throbbing in the head, inability to gain weight, eye disease or blindness, and sleep disturbances at various times, for example difficulty falling asleep, sleeping too much, waking up at night, and falling asleep again. There were other participants who experienced joint or muscle pain, high blood pressure, palpitations, shortness of breath, feelings of pain throughout the body, weakness, dizziness, and gastrointestinal symptoms. For the genocide survivors, these symptoms are triggered by their traumatic memories and are somewhat chronic.

##### 3.2.4.1. Reference to cultural values for body related elements

In most cases, the participants associated the culture of not showing suffering with body-related symptoms. For example, the survivors said that they suffer from headaches and sleeping difficulties when they have problems and think that there is no one to share their suffering. Conversely, body-related symptoms might have a relationship with the culture of ignoring people who are not strong and active. According to the trauma survivors, the lack of community reputation leads to the feeling of loss of vitality, which in turn may provoke too much thinking and body-related disorders. Here, the attitudes of medical staff were mentioned as a factor of chronicity for body-related disorders. For example, the trauma survivors explained how their trauma body-related symptoms were considered and treated for a long time without effect as medical symptoms due to the culture of treating somatically without exploring their relationship with the past traumatic experience.

#### 3.2.5. Changes in family interest and management

##### 3.2.5.1. The desire to give birth to many children

After the traumatic events, such as genocide and war, many families had been exterminated or only few people survived. Those who survived felt alone, with a feeling of having an empty family. Therefore, they are motivated to give birth to many children as a way to regain care, hope, prosperity, and respect in the community. Others want to give birth to many children because they do not know their genealogy and identity and want to be the starting point of their families. Note that there were some survivors who showed that, even though they want to have many children, they do not wish to live with them to prevent them from sudden and subsequent traumatic events that could kill them.

##### 3.2.5.2. Loss of interest in marriage

Some participants shared their view that, since the trauma, they no longer see the importance of marriage. The reason for this perception is that they had witnessed people being betrayed by their partners or parents betraying their children. Others were abandoned by their partners when they were imprisoned, while others were abandoned by their parents. After this experience, they changed their minds and believe that it is better to live alone and not to marry or to give birth to a child in this unjust world without tolerance.

##### 3.2.5.3. Loss of family harmony

The majority of the study participants confirmed that family harmony was lost after the trauma. The participants indicated that they were no longer able to communicate smoothly within the family and help each other and that trust, as it once was, had decreased significantly. Enthusiasm had decreased and the need to be alone had increased significantly. Parents reported being more aggressive toward their children, while partners noted high levels of family conflict. The survivors reported that these family communication difficulties are the factor of continuous family conflict and heart wounds.

##### 3.2.5.4. Difficulties in family resource management

It has been noted that the perceptions associated with trauma can affect the ability and need to manage family resources. Some of the survivors reported that, after the traumatic event, they realized that everything was for nothing and therefore found it absurd to maintain or manage resources. In the same vein, other participants expressed that the proper management of family resources requires a mental capacity, such as that of controlling everything, which they no longer possess due to the trauma. Some individuals who grew up in an orphanage shared that they had difficulty managing the family income well as this is something that is learned by imitating parents and they did not have the opportunity to grow up in a family.

##### 3.2.5.5. Reference to cultural values for changes in family Changes in family interest and management elements

The culture that promotes reproductiveness and prosperity was related to the desire to have many children, while the culture of fatalism and holding onto the sadness was linked to a lack of family harmony, loss of interest in marriage, and difficulties in family resource management. Some participants expressed that, in their culture, people are respected when they have many family members around them. However, many of them had lost their relatives during the traumatic events. This why they were keen to give birth to more children. Conversely, other trauma survivors indicated that they do not have any person to share their problems and that everything is predetermined, which is why they lost interest in marriage and even those who were already married face difficulties in maintaining family harmony.

### 3.3. Cultural scripts elements of the third stage: positive changes

#### 3.3.1. Personal strength: self-determination and increased thirst for phenomenal performance

The participants in this study indicated that life-threatening experiences that severely shook their hearts gave them more courage to strive for a better life. On the one hand, the motivation to work hard stems from the fact that they had to start their lives from scratch after traumatic experiences due to the loss of material possessions and family members and the lack of support. On the other hand, they are determined to succeed because they recognize that they have wasted a lot of time while others have made progress (e.g., former prisoners and former combatants).

In addition, the participants indicated that they like to work hard to correct and compensate for their bad reputation in the past. For example, former prisoners need to work hard and be a good example in the community. Others believe that they must work hard and avoid anything that might put their lives in a critical situation. For example, the survivors of war and genocide try their best not to beg or kneel down before the enemy. They say “utaribwa ntamenya gukinga,” meaning “the one whose property/materials were not stolen cannot know how to close doors,” or “hari ibyo utabonesha amaso atararize,” meaning “there are things you cannot see with eyes that have never cried.”

#### 3.3.2. Increased wisdom

Most participants reported that the unexpected, difficult, and scary moments that they had survived made them wiser. They have become more cautious and think critically before taking actions to avoid serious mistakes or even crimes. They now recognize the importance of information as ignorance endangers lives during a war or genocide. They recognize the need to think twice before saying something because they understand better the harmful consequences of hate speech.

#### 3.3.3. Appreciation for life: an increased sense of gratitude and kindness

The trauma victims believe that many people betrayed them, while others tried their best to save their lives. Many were saved by so-called poor people, but they gave them all the necessities, such as food, drink, and clothing, to save their lives. There were people who had been determined to help others at the risk of their own lives. Therefore, the trauma survivors reported learning from these benevolent and kind people and applying the same legacy to people in need.

#### 3.3.4. Human rights activism

The participants said that their traumatic experiences were marked by injustice, oppression, and discrimination, which violated human rights. This is why they felt responsible for fighting for human rights and against injustice.

#### 3.3.5. Spiritual growth

The trauma survivors, mostly those who were not believers before traumatic events, testified that God is their only savior and that their life could end without him. They believe that they used to neglect the power of God but that now nothing is possible without his blessing hands. They argued that they constantly pray, fast, and meditate to thank God and show him their plan. A significant number agreed that it was only by divine grace that they were healed from the effects of their trauma.

##### 3.3.5.1. Reference to cultural values for positive changes

Religiosity, self-reputation, and social connectedness are the cultural values that the trauma survivors described as being related to post-traumatic growth. They explained that their culture encourages self-reliance attitudes and autonomy to have a good self-reputation. This is why, even after being shaken by traumatic events, they decided to strive for their life to protect their reputation. In addition, it is culturally encouraged to reward people with gratitude and offer mutual support despite life’s challenges, and this social connectedness value helped the trauma survivors to be more grateful and helpful and to fight against injustice more than before. Furthermore, Christianity was found to be related to spiritual growth.

### 3.4. Semi-quantitative analyses

Table 4 shows the analyses of the occurrence of codes in different categories and sub-categories. Cognitive appraisals and changes in interpersonal relationships were the two most often mentioned groupings, whereas changes in family interest and management were mentioned least often. From these data, a matrix of the joined comments was created using MAXQDA (Figure 1), illustrating that the most often mentioned groupings, including cognitive appraisals, worldview, interpersonal relationships, and growth, were closely networked while family interest and management and others were far away from the other groupings in the network. The closeness of the categories is presented in Figure 1 and represents a proxy for a network of every element as this would be too complex to show, consisting of 270 elements.

**FIGU RE 1:**
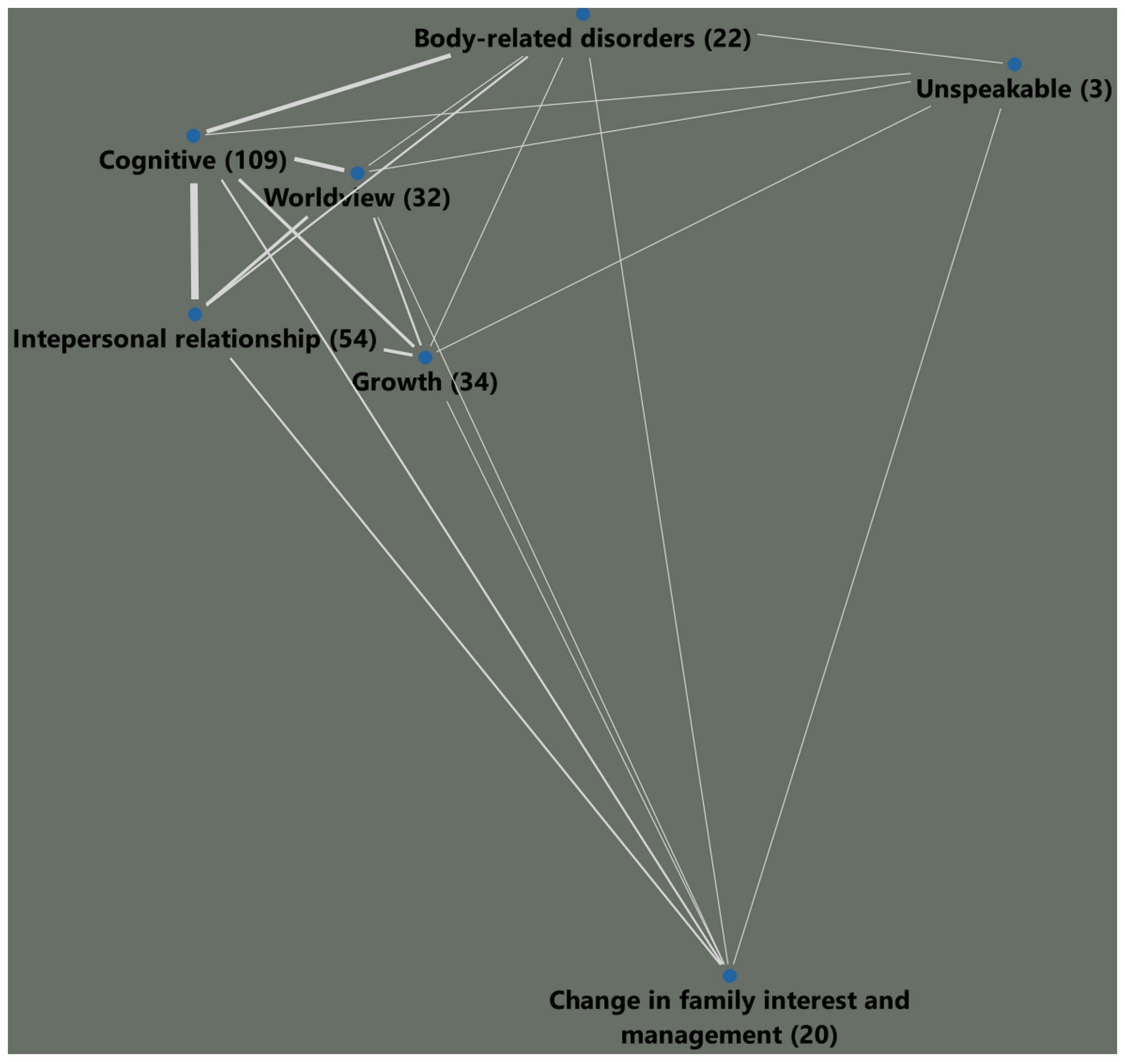
Network of categories according to the participants responses.

## 4. Discussion

This study had the objective of responding to the two following questions: What are the post-traumatic culture-related appraisals, referred to as CSTs elements and their groupings that are expressed by trauma survivors in East Africa? How are the CSTs elements related to East African cultural values as reflected by the participants? The results have revealed that East African trauma survivors exhibit further post traumatic sequalae which are not necessarily found in PTSD criteria but which need clinical attention. Post traumatic appraisals seem to evolve in a process that can be described as stages. The first stage consisted of cultural scripts elements of the unspeakable, which represent the perceptions that survivors are not able to talk about and represent one category of elements. The second stages consisted of heart wounds and painful scars that represent five categories of elements including cognitive, interpersonal relationships, worldview, body related elements and family interest and management elements. The last stage consisted of positive changes or growth elements.

The cultural values that served as a reference consisted of not showing the suffering, Christianity, community reputation, solidarity, social connectedness, social cynicism, and reproductiveness, among others. At this point, cultural values are defined not according to one of the value models established in the literature (e.g., Schwartz, 2014) but in a broader semantic sense as prospective and prescriptive collective beliefs; they affect the ethical behavior of persons in a given cultural region.

As mentioned by the results of this study cognitive and worldview elements have been most frequently described by the participants, followed by interpersonal and family, body, and elements concerning positive change. This corroborates the previous literature that have shown that traumatic experience affect cognitions, beliefs and interpersonal relationships (McCann and Pearlman, 1990; Zoellner and Maercker, 2006; Engelbrecht and Jobson, 2014; Chentsova-Dutton and Maercker, 2019). Furthermore, this study highlighted specific CSTs elements and the cultural values that serve as reference. The indications of cultural values that are related to trauma sequalae is consistent with previous research which have shown that that culture plays a great role in the shaping and manifestation of post-traumatic symptoms and that the current diagnostic taxonomies are not universally applicable (van Rooyen and Nqweni, 2012; Heim et al., 2022).

At the cognitive level, the negative perception of self, feeling of bitterness, feeling of helplessness, and revengeful thoughts were among the cultural script elements identified. The literature has indicated that all of these script elements are mental representations and practices that are still comprehensible but understood as abnormal and undesirable (Chentsova-Dutton et al., 2014) and that they are influenced by culture (Engelbrecht and Jobson, 2020). Nevertheless, this study shed light on survivors’ perception of why these script elements occur in the studied population. The atrocities and shameful acts committed against survivors, devaluation, bullying, feeling of a loss of beauty and reputation, and shame about the cruel crimes committed in the case of former prisoners were the factors associated with this negative self-image and helplessness.

Results of this study have revealed that, the feeling of bitterness was associated with thinking about loss, such as loss of people, loss of a part of the body, loss of property, and loss of lifetime. Previous grief research has indicated a close relationship between bitterness and loss of close person (Maercker et al., 2013; Heeke et al., 2017; Maccallum and Bryant, 2019), but in this case it could be shown that there are other types of loss that can be correlated with a feeling of bitterness. The revengeful thoughts and embitterment were explained by the participants as being induced by the proximity of survivors and perpetrators. Living with a perpetrator reveals feelings of injustice, humiliation, and breach of trust. This result supports the previous literature that indicated that revenge and embitterment are intertwined in the context of traumatization and might be related to post-traumatic symptoms (Gäbler and Maercker, 2011; Linden and Arnold, 2021). Furthermore, this study indicated that the cultural value that prohibits or allows revenge is the trigger of revengeful thoughts and that, when revenge is not possible, it provokes feelings of shame, guilt and bitterness. Further cultural values may be related to cognitive CST elements, including a culture that promotes the value of not showing suffering and maintaining one’s self-reputation.

With regard to the worldview cultural script elements, three kinds were identified that consist of negative changes in the beliefs about the world, hypersensitivity to incidents, and hedonism. Partly, these results corroborate the previous studies that indicated that survivors of trauma perceive the world as less benevolent and predictable (Edmondson, 2014). However, they add to the contextual and cultural lens that induces these changes in this population, including the nature of traumatic events in East Africa, which are usually sudden, multiple, and unpredictable, and the cultural values of Christianity and solidarity. The perception of the nature of traumatic events was, on the one hand, the factor of hedonism as the survivors find no need to plan for the future while living in this unpredictable world. These results corroborate previous studies that indicated that hedonism can mediate the relationship between trauma exposure, the social support process, and the extent of PTSD symptoms (Maercker and Hecker, 2016). On the other hand, the negatives change in the beliefs about the world and hypersensitivity to incidents were associated with the loss of belief in the power of God and the solidarity of people which were the positive cultural values of their community before the traumatic events. These results are in line with the previous studies that indicated that religiosity, and social cynicism might influence adaptive cognitive processing of the traumatic experience (Nalipay et al., 2017).

Regarding the interpersonal cultural script elements, this study yielded both negative and positive CST elements. The negative elements include bragging about achievements, mistrust, and the need for care and consideration, while the positive elements include valorization of personal relationships. The literature has indicated that trauma has an impact on social, interpersonal relationships (Beck et al., 2009; Maercker and Hecker, 2016). However, these results show that traumatic experiences can influence interpersonal relationships, like valorization of others, positively. Furthermore, relevant cultural values, like hypocrisy, social cynicism, and the need for community reputation, were found in the focus groups to be related to negative interpersonal relationships, while social connectedness value was related to positive interpersonal relationships.

Adjacent to the interpersonal cultural script elements are the script elements of family interests and management. Interestingly, this domain tends to shift into behaviorally relevant elements rather than just perceptions of interpersonal interactions. The main element groups include the desire to give birth to many children, loss of interest in marriage, loss of family harmony, and difficulties in managing family resources. The participants testified that culture has a substantial role in the occurrence of these changes. In most African cultures, procreation and having many children are considered to be a sign of pride and wealth (Kokole, 1994; Atake and Gnakou Ali, 2019). However, some families lost many family members during different traumatic events, and they have a feeling of empty and non-respected families (Mutuyimana et al., 2022).

Therefore, trauma victims may have a greater need to have many other children, even though it was not in their plan before traumatic exposure. Conversely, for the large subgroup of genocide survivors in this study, it was very unexpected to be traumatized by a friend, a neighbor, a parent, or another close member of the family. However, this situation happened many times in East Africa as a way of deviating from cultural values and norms. The African cultural values of Ubuntu and Ubumuntu (humanity toward others) and integrity were replaced by deceitfulness (Johannes and K., 2022). Therefore, this deviation was related to loss of trust in and value of people, especially close people. As a result, victims’ survivors find no need to continue living in family harmony (as a value) or become unable to manage their family or make a positive mood in their family.

Body-related changes are an important area of change that has often been described, particularly in the literature on trauma in East Africa (Musisi, 2004; Munyandamutsa et al., 2012) and on post-traumatic stress and somatic symptoms in general (McFarlane Ao and Graham, 2021; Kratzer et al., 2022). The reported script elements include sleep problems, headaches, backache, temporary loss of consciousness, a sensation of throbbing in the head, skin aches, inability to gain weight, and eye disease or blindness, among others. In this study, we intended to shed light on the role of culture in the occurrence of these symptoms. We found that the role of culture is the way in which victims describe their symptoms in their language, which can cause confusion between medical complaints and trauma-related disturbances. For example, feeling throbbing in the head was confused by clinicians with usual headaches. However, the patients wanted to explain that, after the traumatic event, they felt something beating in the brain, similar to what had been used to torture them during the genocide or war. In addition, this study revealed that most somatic symptoms were considered to be normal because they seem to be common in many people and have a temporary character, for example sleeping problems and dizziness.

These symptoms cannot be considered as real symptoms of the trauma but as potentially symptomatizable experiences as the trauma survivors were alarmed to suffer from particular bodily sensations, emotions, thoughts, or other experiences that could endanger their life. However, these symptoms were noticed temporarily; others can be noticed as strange or annoying but without being unduly alarming (Ryder and Chentsova-Dutton, 2015). Other symptoms, such as pain, palpitations, and shortness of breath, however, were reported as frequent symptoms by the interviewees because they are associated with the aforementioned cognitive and interpersonal changes. This is in line with the literature that indicated that cultural scripts for serious distress can make some symptoms constituent and significant (Ryder and Chentsova-Dutton, 2015). Possibly relevant values seem to be the ability to communicate with others and again the reputation within the group (which can also include not communicating these symptoms) and are likely to increase interoceptive sensibility (Ventura-Bort et al., 2021).

Furthermore, the study purposely included positive changes to examine these possible elements of a cultural script as well. Here were found self-determination, a thirst for phenomenal performance, for example “I do not want to fail,” wisdom, for instance “thinking twice before acting,” an increased sense of gratitude and kindness, for example “people are grateful,” and spiritual growth, such as “nothing is impossible for God.” The literature has indicated that adversities can be a source of strength and opportunity for post-traumatic growth. Factors of post-traumatic growth (PTG) include optimism, social support, coping strategies, and personality characteristics (Zoellner and Maercker, 2006). Although cultural contents play an important role in influencing positive changes of individuals after traumatic events, there is a fundamental deficiency in showing how culture influences PTG in non-Western cultures (Kashyap and Hussain, 2018).

Contrary to the belief “what does not kill you makes you stronger,” the East Africans interviewed indicated that their post-traumatic positive changes happened because of the cultural values and beliefs of the need for respect, self-reliance, a culture that promotes a good reputation and integrity, a culture that promotes kindness and gratitude, and a culture of widespread Christianity. For them, in addition to learning from their past suffering, their positive changes were motivated by cultural values and the need to regain a positive societal image (Slade et al., 2019).

Finally, they are unspeakable cultural scripts elements. These elements consisted of the first level of post traumatic appraisals. Although trauma survivors believed that talking about their trauma is an initial step toward healing but, still some of post traumatic appraisals were indescribable for them. Three reasons were explained. First, the trauma survivors do not have the words to explain their current sufferance. Second, fear of negative feedback or humiliation. Third, perceived experiences are beyond culturally accepted words, e.g., talking in details about sexual violence. According to the trauma survivors, unspeakable post traumatic appraisals are correlated with many psychosocial problems. These results corroborate with previous studies that indicate that dysfunctional posttraumatic cognitions, disclosure attitudes, and social disapproval correlate positively with PTSD severity (Mueller et al., 2008). Survivors have indicated that these unspeakable appraisals attain slowly or rapidly further stages depending on social, clinical and political support and acknowledgement. This is also in line with the previous study that have indicated that personal barriers to disclosure are negatively correlated with social support and positively correlated with post traumatic deprecation (Nordstrand et al., 2020). However, this study shed light on the factors that push the survivors to not be able to share their post traumatic experiences and indicated the levels involved in the perception of traumatic experiences. The cultural value of holding sadness and community reputation were related to unspeakable CST’ elements.

The remaining semi-quantitative results show that, at the level of script categories, a tentative network of signs and symptoms obtained by analyzing the similarities of codes in categories, which is actually a prerequisite for cultural scripts, makes only limited sense. Here, all the mental categories, including cognitive, worldview, interpersonal relationships, body-related changes, and growth, cluster closely together and the more behavioral category of changes in family interest and management is less connected to it. This suggests that there are two modes of perception: “what do I think?” and “how do I act?” It would also have been possible for the script elements of interpersonal relationships to be more closely related to those of family interest and management in a more holistic way. Here, sub-category-level or element-level investigations using a quantitative study should follow to examine just these connections as well.

Generally, this study has indicated that post-traumatic sequelae affect the entire psychosocial and cultural lives of the survivors. Trauma can change the cognitive way of thinking, worldview, and interpersonal relationships, which in turn can provoke body-related symptoms (Epstein, 1991; Chentsova-Dutton and Maercker, 2019). Furthermore, this study is in line with the previous research that has indicated the possibility of cohabitation between negative and positive traumatic appraisals (Zoellner and Maercker, 2006). Interestingly, this study has revealed specific CST elements that includes change in family interest and family management. This might be related to the importance given to the family in a collective community. In addition, most traumatic experiences have reference to cultural values, and the participants indicated that some CST elements were for them more pathological, even though their assessment does not fall into any mental health category of the disorders.

### 4.1. Study limitations

This study is subject to several limitations. The most notable problem is that the cultural script theory actually seeks to identify temporal relationships between script elements (Chentsova-Dutton and Maercker, 2019; Chentsova-Dutton and Ryder, 2019). Due to the underlying complexities of the study reported here for the identification of elements alone, this more advanced step had to be discarded for now. However, one merit of the study is that it elaborated on the references to cultural values (see Maercker et al., 2019). In addition, the cultural script theory implies that conclusions are only made at the element level. However, for the purposes of preparatory work, the current analysis started by grouping the elements into sub-categories. Whether further studies continue with the 270 individual elements or the 27 subcategories remains to be decided.

In addition, there are various methodological limitations. First, this is a retrospective study that asks about the experience of past exposure to traumatic events, and it might be subject to memory bias. Even though we tried to include experts and trauma survivors from different countries, the majority of the trauma survivors were from Rwanda. Future studies should include more trauma victims from other East African countries. The recording of interviews was not allowed. We tried to mitigate this limitation by using multiple data collectors and conducting a debriefing of data collectors for every session, but some content might have been omitted. At this stage, with only qualitative results, it is not possible to know the cultural scripts of trauma as the results do not yet highlight the relationships between the scripts’ identified content and the sequence. Therefore, future quantitative and longitudinal studies are recommended.

## 5. Conclusions and implications

Despite the limitations, this study has several implications. It is the first study to highlight cultural script elements of trauma sequelae in East African countries. This contributes substantially to the existing literature and creates an avenue for research and clinical practices. First, the cultural scripts elements mentioned by the trauma survivors and experts in the domain of psychotherapy did not necessarily fulfill the criteria of trauma-related disorders, but many participants reported that they were a huge health condition burden that required help. Therefore, it is of great importance to clinicians to consider these elements of cultural scripts in their interventions and not only to be guided by the categorization of mental disorders. It is a fact that the use of mental health services in the affected traumatized groups, such as those in Rwanda, is very low (Kayiteshonga et al., 2022). If trauma-related changes closer to the experience of the affected persons are captured, then this can be a starting point for the use of such services. Second, this research might be an entry point for future studies on the temporal relationships of cultural script elements in this sample and in different cultures. Third, there are the factors of cultural values, which—as the results show—are related to cultural scripts. The cultures of holding the sadness, Christianity, and hypocrisy were mentioned as being related to cultural script elements, among others. Therefore, it is highly important for clinicians and policy makers to help trauma victims break their silence and reestablish their trust. Community awareness and culturally adapted interventions are needed to help trauma survivors use their positive changes to recover from their negative and harmful changes.

## Data availability statement

The raw data supporting the conclusions of this article will be made available by the authors, without undue reservation.

## Ethics statement

The studies involving humans were approved by University of Rwanda College of Medicine and Health Sciences Ethics Committee. The studies were conducted in accordance with the local legislation and institutional requirements. The participants provided their written informed consent to participate in this study.

## Author contributions

All authors listed have made a substantial, direct, and intellectual contribution to the work and approved it for publication.

## Appendix

Interview guide questions

**Participants**

1. What are the changes/appraisals* that happened to you after your exposure to genocide, war, imprisonment, or childhood trauma (depending on the group)?
2. What other changes/appraisals (negative) that occurred as a result of a traumatic experience are not listed here?
3. What positive changes/appraisals (growth) occurred in you after the trauma?
4. What are the cultural values that you can refer to the above-mentioned changes/appraisals?

**Experts**

1. What are the changes/appraisals* that you observe in your clients as results of trauma exposure?
2. What other changes/appraisals (negative) do you find in your clients or in your community? Please comment on each.
3. What other positive (growth) changes do you find in your clients or in your community? Please comment on each.
4. What are the cultural values that you can refer to the above changes/appraisals?

We used negative or positive changes to make the questions clearer to the participants. *The Kinyarwanda term for appraisal is *impinduka*.

## Abbreviations

CSTs: Cultural scripts of trauma
PTG: Post-traumatic growth
